# Intraabdominal hypertension is less common than it used to be: A pilot step wedge trial

**DOI:** 10.2478/jccm-2025-0002

**Published:** 2025-01-31

**Authors:** Shane Smith, Fran Priestap, Neil Parry, Robert Arntfield, Patrick Murphy, Kelly Vogt, Ian Ball

**Affiliations:** Office of Academic Military Medicine, Western University, London, Ontario, Canada; Department of Surgery, Western University, London, Ontario, Canada; London Health Sciences Centre Trauma Program, London, Ontario, Canada; Department of Medicine, Western University, London, Ontario, Canada; Department of Surgery, Division of Trauma and Acute Care Surgery, Medical College of Wisconsin, Milwaukee, WI, USA

**Keywords:** intra-abdominal hypertension, abdominal compartment syndrome, resuscitation, shock, guidelines

## Abstract

**Objective:**

This is a pilot study to determine the feasibility of a multicentre stepped wedge cluster randomized trial of implementing the 2013 World Society of the Intraabdominal Compartment Syndrome (WSACS) guidelines as an intervention to treat intraabdominal hypertension (IAH) and abdominal compartment syndrome (ACS) in critically ill patients.

**Design:**

Single-centre before-and-after trial, with an observation / baseline period of 3 months followed by a 9-month intervention period.

**Setting:**

A 35 bed medical-surgical-trauma intensive care unit in a tertiary level, Canadian hospital.

**Patients:**

Recruitment from consecutively admitted adult intensive care unit patients.

**Intervention:**

In the intervention period, treatment teams were prompted to implement WSACS interventions in all patients diagnosed with IAH.

**Measurements and Main Results:**

129 patients were recruited, 59 during the observation period and 70 during the intervention period. Only 17.0% and 12.9%, respectively, met diagnostic criteria for IAH. Many recruited patients did not have intraabdominal pressures measured regularly per study protocol. There was no difference in ICU mortality for patients in either cohort or between those with and without IAH.

**Conclusions:**

The incidence of IAH in our patient population has decreased significantly since 2015. This is likely due to a significant change in routine care of critically ill patients, especially with respect to judicious goal-directed fluid resuscitation. Patient recruitment and protocol adherence in this study were low, exacerbated by other staffing and logistical pressures during the study period. We conclude that a larger multicentre trial is unlikely to yield evidence of a detectable treatment effect.

## Introduction

Intraabdominal hypertension (IAH) is defined as a “sustained or repeated pathological elevation in intraabdominal pressure of greater than or equal to 12 mm Hg”; abdominal compartment syndrome (ACS) is defined as IAH of equal to or greater than 20 mm Hg that is associated with new organ dysfunction/failure. [1]. IAH is very common in intensive care unit (ICU) patients, although its actual prevalence may be underestimated [2,3,4,5].

In a previous study conducted by our group in 2015, we investigated the prevalence and incidence of IAH in our level 1 trauma, medical, and surgical ICU by obtaining twice-daily intraabdominal pressure measurements on 285 consecutively admitted patients [2]. Results showed that 30% had IAH at admission and another 15% developed IAH during their admission to the ICU; 3% of patients developed ACS. These rates were higher than previously thought, and higher than would otherwise have been appreciated in the absence of universal screening. In this study, predictors of IAH were obesity, sepsis, mechanical ventilation, and greater than 3 L fluid balance at 24 hours. IAH was found to be an independent predictor of mortality. Furthermore, mortality was shown to increase with increasing grades of IAH. These findings underscored the importance of identification, prevention, and treatment of IAH in all critically ill patients.

The 2013 World Society of the Abdominal Compartment Syndrome (WSACS) clinical practice guidelines [1] provide consensus definitions, as well as an approach to identifying patients whose intraabdominal pressures should be measured and monitored. They recommend protocols be implemented to prevent sustained IAH in critically ill patients. (Table 1) Their algorithms include serial pressure measurements, medical treatment options, and when to consider decompressive laparotomy. The medical management algorithm addresses five contributors to the pathophysiology of increased intraabdominal pressure: 1) evacuate gastrointestinal intraluminal contents; 2) evacuate intraabdominal space-occupying lesions; 3) improve abdominal wall compliance; 4) optimize fluid administration; and 5) optimize systemic/regional perfusion. All of these potential contributors can be addressed simultaneously, and within each category, a step-wise approach from least to most invasive intervention is provided (see Methods section for details). The WSACS acknowledges a “lack of high-quality evidence to base decision-making” but suggests that “these guidelines should be used as guides for any institution or clinician to initiate their care of the critically ill.”

**Table 1.** Medical management algorithm for management of IAH/ACS, adapted from World Society for the Abdominal Compartment Syndrome

**Step Number / Action**	**Evacuate gastrointestinal intraluminal contents**	**Evacuate intra-abdominal space-occupying lesions/collections**	**Improve abdominal wall compliance**	**Optimize fluid administration**	**Optimize systemic/regional perfusion**
Step 1	Insert nasogastric and/or rectal tube	Abdominal ultrasound to identify lesions	Ensure adequate sedation and analgesia	Avoid excessive fluid resuscitation	Goal-directed fluid resuscitation
	Initiate prokinetic agents		Remove constrictive dressings, abdominal eschars	Aim for zero to negative fluid balance by day 3	

Step 2	Minimize enteral nutrition	Abdominal computed tomography to identify lesions Percutaneous catheter drainage	Consider reverse Trendelenberg position	Resuscitate using hypertonic fluids, colloids Fluid removal through judicious diuresis once stable	Hemodynamic monitoring to guide resuscitation
	Administer enemas				

Step 3	Consider colonoscopic decompression	Consider surgical evacuation	Consider neuromuscular blockade	Consider hemodialysis/ultrafiltration	
	Discontinue enteral nutrition				

Step 4	If intraabdominal pressure greater than 20 mm Hg and new organ dysfunction/failure is present, patient's IAH/ACS is refractory to medical management. Strongly consider surgical abdominal decompression.				

Adapted from WSACS guidelines.

Based on our prior work and other published evidence linking IAH with ICU mortality [6], we decided the next step was to investigate whether targeted implementation of the WSACS interventions would lead to improved outcomes for critically ill patients. The nature of the guideline-suggested interventions clearly precludes blinding of treating physicians. Additionally, because many of these interventions are relatively common in the ICU population anyway, it could be very easy for treatment creep to occur within a given unit, wherein patients in the control arm are treated increasingly like the treatment arm participants, diminishing any signal of intervention benefit. To mitigate these issues, we determined that a stepped-wedge cluster randomized trial would be an appropriate study design, in which clusters (multiple different ICUs) would be sequentially transitioned from an observational to an interventional period in a randomized order [7].

The present pilot trial was designed to determine the feasibility of a multicentre stepped wedge cluster randomized trial, including assessment of patient recruitment, adherence to protocols, and identification of other trial barriers. Specifically, we sought to observe differences in a single tertiary care institution's (meaning a hospital affiliated with a university that acts as a referral centre for other community hospitals) ICU during a three-month baseline observation period and then a nine-month intervention period with implementation of WSACS guidelines for critically ill patients.

## Methods

A pilot feasibility trial was conducted in the ICU at Victoria Hospital in London, Ontario, Canada. Victoria Hospital is a Level 1 trauma centre with a referral base of 2 million people. The ICU is a 35-bed medical-surgical-trauma unit. The observation period was three months, followed by an intervention period of nine months. All adult patients (>/=18 years) admitted to the ICU were assessed for trial inclusion. Exclusion criteria included lack of consent, pregnancy, and cases where the clinical team were unwilling to enroll a patient for any reason (examples might include expectation of improvement without intervention, opposition to the WSACS guidelines, or any other reason). Recruitment began in January 2021 and finished in March 2022. Ethics approval for “Intra-Abdominal Hypertension Study” was obtained January 9, 2020 from the Health Sciences Research Ethics Board at Western University (#113592). All procedures were followed in accordance with the ethical standards of the responsible committee on human experimentation and with the Helsinki Declaration of 1975. The study was approved with a deferred consent model, where patients were enrolled at admission and consent for trial continuation and data collection occurred within (48) hours.

The primary outcome for this pilot study was trial feasibility. Feasibility metrics included: recruitment rates, consent rates, protocol violations, and identification of other trial barriers. Secondary outcomes were the incidence of IAH and mortality. Patients were defined as having IAH if they had two or more consecutive measurements of intraabdominal pressure greater than or equal to 12 mm Hg, as measured twice daily during the first five days of their ICU admission. These bladder pressure measurements were conducted at 10:00 and 22:00 by the ICU bedside nurses. Pressures were measured via the bladder using the modified Kron technique described elsewhere [2] and endorsed by the WSACS [1]. During the intervention phase, clinical teams whose patients were diagnosed with IAH were prompted by the research team to implement WSACS guidelines [1]. Specific interventions recommended by these guidelines are summarized in Table 2; refer to WSACS guidelines for full details please. During the observation phase, the research team merely noted the clinical team's response to any elevated bladder pressure measurements but did not intervene or prompt the clinical teams.

**Table 2.** Feasibility Metrics

**Population**	**n**
**Patients meeting inclusion criteria**	114
**Exclusion criteria** -- death prior to assessment	5
Pregnancy	0
Expected ICU discharge less than 24 hrs.	8
Organ donors	0
Clinical team or SDM declined to enroll	13
Enrolled by deferred consent but subsequently declined	2
No SDM available within the time constraint	18
**Total number enrolled**	70

There were no documented protocol violations. As per REB, consent was not required for the 3 month run-in observational phase.

Patient data collected included age, sex, multi-organ dysfunction score (MODS)[8], intraabdominal pressure measurements, details of clinical status and management, ICU mortality, and in-hospital mortality.

## Results

Only 129 patients were recruited; 59 during the observation period and 70 during the intervention period. During the observation period (January 7–March 11, 2021) there were a total of 220 ICU admissions. During the intervention period (June 14, 2021–March 13, 2022) there were a total of 1043 ICU admissions. See reasons for non-recruitment in Table 2. There were no cases where the clinical teams refused to enroll eligible patients.

Patient demographic and clinical characteristics were similar across periods in terms of age (overall average age 61.6 years), sex (overall 58.8% male), and medical/surgical/trauma indication for admission to ICU. The average MODS was slightly higher for patients in the intervention period, 5.5 versus 4.1 (p-value 0.003) (Table 3).

**Table 3.** Patient demographics and clinical characteristics

**Descriptor**	**Total**	**Observation period**	**Intervention period**	**p-value**
N	129	59	70	
Male, N (%)	76 (58.8)	33 (55.9)	43 (61.4)	0.592
Age, mean ± s.d., years	61.6 ± 14.5	60.6 ± 15.4	62.7 ± 13.7	0.672

Indication for admission, N (%)				0.542
Medical	90 (69.8)	44 (74.6)	46 (65.7)	
Surgical	15 (11.6)	6 (10.2)	9 (12.9)	
Trauma	24 (18.6)	9 (15.3)	15 (21.4)	
MODS, mean ± s.d.	4.8 ± 2.8	4.1 ± 2.3	5.5 ± 2.9	0.003

s.d., standard deviation; MODS, multi-organ dysfunction score; p-values are for Fisher's exact test for 2 proportions, t-test for comparison of means, and Pearson chi-square test of association for categories

In terms of protocol adherence, of the 129 patients recruited, a significant number did not have intraabdominal pressures measured during their first five days of admission to ICU. This was true in the observation period (32.2% of patients did not have at least two measurements) and was worse in the intervention period (48.6%; although not statistically significantly different with p=0.073). Overall 26.4% of patients did not have a single intraabdominal pressure recorded, and 41.1% did not have at least two measurements. There was no correlation between the level of the first recorded intraabdominal pressure and whether or not a second pressure was obtained (p-value 0.331).

Overall, 21 patients met our definition of IAH (intraabdominal pressures of 12 mm Hg or greater measured on two consecutive occasions), for an overall prevalence of 16.2%. This included 11 patients during the observation period (8.5%) and 10 patients (7.8%) for the intervention period. Using a more liberal definition of “repeated” (not necessarily consecutive) measurements of at least 12 mm Hg, the respective prevalences were 22.0% and 27.1%, respectively. Please see Table 4. Only one patient had two measurements of 20 mm Hg or greater (in the intervention phase).

**Table 4.** Prevalence of IAH, ACS, and incomplete IAP measurements

**Descriptor**	**Total**	**Observation period**	**Intervention period**
IAH: IAP ≥ 12 mm Hg on two consecutive measurements (N (%))	21 (16.2 )	11 (8.5 )	10 (7.8 )
IAP ≥ 12 mm Hg on any two measurements (N (%))	32 (24.8)	13 (22.0)	19 (27.1)
IAP ≥ 20 mm Hg on any two measurements (N (%))	1 (0.8)	0 (0.0)	1 (1.4)
Patients who did not have IAP measured twice (N (%))	53 (41.1)	19 (32.2)	34 (48.6)

IAH, intraabdominal hypertension; ACS, abdominal compartment syndrome; IAP, intraabdominal pressure; p-values are for Fisher's exact test of two proportions

Further statistical analysis was limited given the unexpectedly low prevalence of IAH (less than half the rate detected in our previous study in the same ICU in 2015)[2]. There was no significant difference in ICU mortality between the observation and study periods (28.8% compared with 28.6%, p = 1.000). IAH (as defined by two consecutive measurements of at least 12 mm Hg) was not associated with ICU mortality (Pearson chi-square p = 0.890). Neither maximum nor mean fluid balance was found to predict a diagnosis of IAH (p = 0.192 and p = 0.224, respectively; data not shown). The median positive fluid balance at 24 hours was 500 mL, with only 7 patients total having a median fluid balance of over 3 L at 24 hours.

The most frequently noted WSACS interventions administered to patients in both cohort groups were diuresis and nasogastric or orogastric intubation with suction, which did not differ between the groups (data not shown).

## Discussion

This pilot trial suggests that a multicentre stepped wedge cluster trial is not feasible.

First, rates of IAH were much lower than those observed in our 2015 data [2]. Overall, only 14.7% of our study cohort had at least two consecutive intraabdominal pressure measurements of 12 mm Hg or higher; this was the same definition used in the previous study in the same ICU, in which we found a prevalence of 30%.

The reasons for the observed decrease in this previously common ICU complication are likely multifactorial. As mentioned in the introduction, many of the “interventions” suggested in the 2013 WSACS [1] are commonplace in the contemporary ICU, suggesting the possibility of a “treatment creep” in which WSACS principles are increasingly part of routine ICU care (including the care of patients in our observation cohort). There is now a tendency toward less aggressive fluid resuscitation [9,10,11], and resuscitation tends to be more carefully guided by point-of-care ultrasound [12] and hemodynamic parameters. In comparison with our 2015 data, wherein we reported a median 24-hour positive fluid balance of 1640 mL, in the present cohort this was 500 mL, with only rare patients in more than 3 L positive fluid balance at 24 hours. ICU care has evolved in a way that seems likely to prevent the development and persistence of IAH. Decompressive laparotomies have anecdotally become very rare in our critically ill patients as a result (none reported in this study cohort, as compared with four in our previous study of approximately twice the number of patients).

This global change in practice has mitigated some of the theoretical gains achievable by uniform adoption of WSACS guidelines. Viewed as an intervention on its own, the WSACS algorithm's treatment effect was likely diminished.

A second potential contributor to the low detection rate of IAH in this study was the relatively low adherence to the requested twice-daily measurement of intraabdominal pressures via bladder catheter. The timing of both observation and intervention phases of this study was unfortunate. The trial period was during peak COVID-19 in our hospital and there were many logistical limitations to running an ICU-based study. Clinical staff were facing many novel challenges in providing routine critical care, and research staff had no access to the unit to provide education about the trial, its methods, and its purpose. It is likely that these competing priorities may have likewise reduced adherence in the intervention phase as well.

This ties into the other main challenge for this study, which was patient recruitment and consent. In our previous study in the same setting, we had been able to recruit nearly 300 patients among almost 400 admitted to the ICU during a four-month period. In the current trial, we recruited less than half of the recruited patients over an (interrupted) 12-month study period. The present study's application to the research ethics board for waived consent was unsuccessful, which posed major recruitment and consent challenges in an environment where neither research staff nor family members / substitute decision-makers had access to the ICU. In fact, trial recruitment was halted several times for these reasons (hence the planned study period of 12 months stretched out longer than one year).

While some of this pilot study's challenges could be mitigated by a repeat attempt with improved patient recruitment and better adherence to the trial protocol (in terms of detecting IAH), we theorize that the evolution of ICU care, and in particular improved judicious fluid resuscitation, mitigates some of the theoretical treatment effect that might have been detected by a similar study even ten years ago. The routine application of more WSACS-type interventions has led to a clinical situation in which interventions applied to a “control” and “treatment” group are increasingly indistinguishable, which is very positive for critically ill patients.

## Conclusion

This pilot study indicates the infeasibility of a multi-center cluster randomized study. The barriers encountered in conjunction with a smaller predicted treatment effect limits the usefulness of a larger trial. The general principles espoused by the WSACS are likely a reasonable approach to preventing and treating increased intraabdominal pressure in ICU patients.
